# A cross-sectional primary care study of knowledge, attitudes, and practices of type 2 diabetes self-care and their association with sociodemographic and sociocultural factors in Cali, Colombia

**DOI:** 10.3389/fpubh.2025.1719863

**Published:** 2026-01-12

**Authors:** Janeth C. Gil, Luis Felipe Ramírez Otero, Gloria A. Tunubala, Clemente Caicedo, Jhan S. Saavedra T., H. A. Nati-Castillo, Juan S. Izquierdo-Condoy

**Affiliations:** 1 Facultad de Salud, Universidad Santiago de Cali, Cali, Colombia; 2 Facultad de Ciencias Básicas, Universidad Santiago de Cali, Cali, Colombia; 3 Servicio de Consulta Ambulatoria de Medicina Preventiva, Red de Salud del Norte E.S.E, Cali, Colombia; 4 Grupo de Investigación en Educación y Salud (GINEYSA), Facultad de Salud, Universidad Santiago de Cali, Cali, Colombia; 5 Grupo de Investigación en Biomateriales y Biotecnología - BEO, Facultad de Salud, Universidad Santiago de Cali, Cali, Colombia; 6 One Health Research Group, Universidad de las Américas, Quito, Ecuador

**Keywords:** Colombia, knowledge, attitudes and practices (KAP), primary care, self-care, type 2 diabetes mellitus

## Abstract

**Introduction:**

Type 2 diabetes mellitus (T2DM) represents a major and rising clinical and economic challenge in Latin America. Strengthening patients’ knowledge, attitudes, and practices (KAP) is essential to improve self-care behaviors and optimize primary care outcomes.

**Objectives:**

To assess self-care KAP among adults with T2DM in Cali, Colombia, and to examine their associations with sociodemographic and sociocultural factors.

**Methods:**

A cross-sectional observational study was conducted among adults (≥18 years) with confirmed T2DM enrolled in a public primary-care network in Cali (January 2020–June 2024). Data were obtained through a 43-item structured questionnaire comprising the culturally adapted DKQ-24 (knowledge), Likert-type items (attitudes), and an adapted SDSCA incorporating traditional-medicine practices. KAP scores were categorized *a priori*. Descriptive statistics were used to characterize the sample, and chi-square tests examined associations between sociodemographic/sociocultural variables and KAP levels.

**Results:**

Of 336 participants, most were women (62.2%) and older adults (≥60 years, 65.7%). High knowledge was observed in 81.8%, positive attitudes in 99.0%, and frequent practices in 88.1%. Gaps emerged in daily meal planning (26.5% correct) and understanding the value of blood-glucose self-monitoring (31.8% correct). Strong behaviors included medication adherence and foot care (both 76.0% frequent); risk behaviors were uncommon (recent smoking 4.0%; dessert/sweet intake 19.0%). Knowledge was significantly associated with socioeconomic stratum (*p* = 0.003), with lower performance at extreme strata. Attitudes differed by marital status (*p* = 0.001), religion (*p <* 0.001), socioeconomic stratum (*p <* 0.001), and household size (*p <* 0.001), showing lower positivity among widowed individuals and households with more than five members.

**Conclusion:**

Adults with T2DM in this urban primary-care setting demonstrate a favorable KAP profile, though persistent gaps remain in dietary planning and glycemic self-monitoring. Targeted, culturally sensitive educational strategies—integrating family and faith-based networks—should prioritize these areas while reinforcing adherence and foot care. Multivariable and longitudinal analyses are recommended to deepen understanding and evaluate tailored interventions.

## 1 Introduction

Type 2 diabetes mellitus (T2DM) has become one of the chronic diseases with the greatest epidemiological and economic impact worldwide (1). According to the World Health Organization, its prevalence continues to rise, especially in low- and middle-income countries, where nearly 80% of people with diabetes are projected to reside by 2025 (2–4). In Latin America, this increase has been particularly rapid, and in Colombia an estimated 7–9% of adults live with T2DM, with a substantial proportion remaining undiagnosed (5–7).

In Colombia, the prevalence of diabetes in adults hovers around 8–9%, with a sustained increase in urban areas. In Cali, records show a notable rise, with reported cases increasing from 6,429 in 2017 to more than 9,200 in 2018. The Valle del Cauca region has rates higher than the national average, driven by obesity, sedentary lifestyles, and social inequality (8, 9).

Diabetes is a metabolic disorder in which the action of insulin fails, producing a persistent elevation of blood glucose. This leads to mitochondrial dysfunction, sustained inflammation, vascular endothelial damage, and progressive deterioration of the kidneys, retina, peripheral nerves, and heart, with serious and multisystemic clinical consequences (10–12).

Diabetes precipitates a spectrum of complications that extends beyond traditional microvascular and macrovascular damage. It manifests as immunometabolic dysfunction with vulnerability to severe infections, energy myopathy, autonomic neuropathy with silent cardiovascular instability, progressive cognitive decline, pancreatic fibrosis, primary diabetic cardiomyopathy, and a pro-inflammatory phenotype that potentiates cancer, systemic frailty, and multi-organ collapse in response to acute stress (13, 14).

Complications arising from poor glycemic control—retinopathy, nephropathy, and neuropathy—increase morbidity and premature mortality while deepening the social and financial burden on health systems (10–12). Among the main challenges to achieving effective control is therapeutic adherence: a considerable fraction of individuals with T2DM experience lapses in treatment continuity, limiting clinical effectiveness. This situation has driven the integration of health-education strategies that consider patients’ knowledge, attitudes, and practices (KAP) related to self-care, which are recognized as key determinants of clinical course and intervention success (15–17).

Regional evidence reinforces this need. In Honduras, the situation regarding diagnosis and treatment is worrisome: nearly half of patients were not registered and did not receive timely care (18). In Brazil, studies in primary care report low levels of knowledge and low levels of knowledge and suboptimal attitudes toward diabetes self-care, including limited perceived importance of lifestyle recommendations and difficulties translating intentions into day-to-day behaviors, among people with T2DM; only about 38% demonstrated adequate self-care knowledge, with notable gaps in foot care among older adults (19). In addition, the family environment plays a critical role: multiple analyses show that family support decisively influences adherence and the performance of self-care practices (20).

Traditional medicine has also attracted growing interest in the region. In Ecuador and elsewhere in Latin America, “plant insulin” (Costus igneus) is popularly recognized for hypoglycemic properties; although experimental studies indicate significant reductions in blood glucose, robust clinical evidence is still lacking (21, 22). Taken together, these antecedents underscore the relevance of exploring non-conventional practices and explicitly linking social, educational, and cultural factors to KAP levels among people with T2DM (23).

Despite the magnitude of the problem, Colombia has limited evidence that integrally characterizes self-care KAP in T2DM and its association with sociodemographic and sociocultural factors, including family support and the use of traditional medicine in primary-care settings. Therefore, the objective of this study was to evaluate self-care knowledge, attitudes, and practices among adults with T2DM in Cali, Colombia, and to analyze their association with sociodemographic and sociocultural factors.

## 2 Materials and methods

### 2.1 Study design and setting

We conducted a cross-sectional observational study using primary data obtained through structured face-to-face and telephone interviews with adults diagnosed with T2DM. These interviews generated first-hand information on sociodemographic characteristics and KAP related to diabetes self-care. Additionally, secondary data from institutional clinical records were used to verify diagnosis, eligibility, and program enrollment.

The study was implemented in the Red de Salud del Norte E. S. E., a first-level public primary care network in northern Cali that was created as part of the decentralization process of the Municipal Public Health Secretariat (Agreement 106, December 29, 2003), and served as the operational setting for participant recruitment and data collection. This integrated description of design and setting contextualizes both the epidemiological structure of the study and the real-world healthcare environment in which participants routinely receive chronic disease management.

### 2.2 Population and sample

The target population comprised users with a confirmed diagnosis of T2DM who were enrolled in the network’s institutional risk-control program between January 2020 and June 2024. Inclusion criteria were: age ≥18 years, residence in Cali (Colombia), enrollment in the institutional program, and provision of written informed consent. Exclusion criteria were: type 1 diabetes, serious comorbid conditions (e.g., cancer, chronic kidney disease, or severe mental disorders), and refusal to participate. Data were collected through scheduled face-to-face or telephone interviews conducted at first-level care points within the network.

### 2.3 Sample size calculation

The total number of users with T2DM in the network was *N* = 3,167. For quantitative sampling, the eligible sampling frame consisted of *N* = 1,465 active records meeting inclusion criteria. The sample size was estimated for simple random sampling (design effect = 1) with a 95% confidence level (Z = 1.96), expected proportion *p* = 0.50 (maximum variance), and absolute precision *e* = 0.05. Applying the finite population correction to the eligible frame (*N* = 1,465) yielded a minimum required sample of 305 participants. To enhance precision and accommodate potential nonresponse/operational losses, we defined an operational sample size of 336, which corresponds to the number ultimately surveyed.

Response rate. The number of individuals contacted was not recorded separately; as a reference, the completion proportion relative to the sampling frame was 336/1,465 = 22.9%.

### 2.4 Data measurement and questionnaire

A structured 43-item questionnaire was administered, organized into four modules: (1) sociodemographic characteristics; (2) knowledge about T2DM; (3) attitudes toward disease management; and (4) self-care practices.

- Knowledge was assessed with a culturally adapted version of the Diabetes Knowledge Questionnaire (DKQ-24) previously validated in Latin American settings (24, 25).
- Attitudes were measured using Likert-type items based on KAP studies in populations with chronic diseases (26, 27).
- Practices were evaluated with an adapted Summary of Diabetes Self-Care Activities (SDSCA), incorporating items on non-conventional practices, including the use of medicinal plants (e.g., “plant insulin”) (28, 29)

Instrument validation included a pilot test with 10 randomly selected users. Internal consistency was assessed with Cronbach’s alpha in SPSS v29.0 (IBM Corp., Armonk, NY), yielding coefficients >0.7 across the three domains.

### 2.5 Variables and data management

Sociodemographic and sociocultural variables were self-reported following standard categories used in the Colombian health system. In particular, socioeconomic stratum was classified according to current Colombian regulations, self-reported in strata 0–5. Other variables (e.g., marital status, schooling, family/social support) were recorded using routine categories from cardiovascular risk and chronic-disease programs, without additional reclassification for this analysis.

KAP domain responses were numerically coded and categorized using predefined cutoffs:

- Knowledge: low (0–18 points), medium (19–29), high (30–32).
- Attitudes: negative (0–13), indifferent (14–26), positive (27–39).
- Practices: infrequent (1–13) vs. frequent (14–26).

### 2.6 Statistical analysis

Descriptive analyses (absolute and relative frequencies) were performed for categorical variables. Associations between sociodemographic/sociocultural variables and the levels of knowledge, attitudes, and practices of T2DM self-care were evaluated using the chi-square test. A *p*-value <0.05 was considered statistically significant. All analyses were conducted in SPSS v29 (IBM Corp., Armonk, NY, USA).

### 2.7 Ethical considerations

The study was classified as minimal risk under Resolution 8,430 of 1993 (Ministry of Health of Colombia) and conducted in accordance with the Declaration of Helsinki. Confidentiality was ensured through coded identifiers and secure data storage. The protocol and informed-consent procedures were approved by the Ethics Committee of Universidad Santiago de Cali and the Ethics Committee of the Red de Salud del Norte E. S. E.

## 3 Results

### 3.1 Sociodemographic and sociocultural characteristics

A total of 336 individuals with T2DM were included; 62.2% were women. Older adults predominated (≥60 years: 65.7%; 60–69 = 34.2%; 70–79 = 23.8%). Regarding marital status, single participants were most frequent (42.6%). Religious affiliation was mainly Catholic (69.3%), followed by non-Catholic Christian (21.4%). Socioeconomically, most participants belonged to strata 2 (51.2%) and 3 (24.7%). Basic education predominated (incomplete/complete primary 56.5%). Living with family was common (85.7%); 11.3% lived alone, and households of one or two cohabitants were frequent (22.9 and 21.1%, respectively). The relationship with cohabitants was mostly rated good (80.4%). Only 12.8% reported membership in a social group, while 71.4% reported receiving support for treatment/illness (Table 1).

**Table 1:** Sociodemographic and sociocultural characteristics of participants with T2DM (*n* = 336).

Variable	Category	*n*	%
Gender	Male	127	37.8
	Female	209	62.2
Age (years)	30–39	7	2.1
	40–49	19	5.7
	50–59	89	26.5
	60–69	115	34.2
	70–79	80	23.8
	80–89	23	6.8
	90–99	3	0.9
Marital status	Single	143	42.6
	Married	90	26.8
	Cohabiting	50	14.9
	Widowed	49	14.6
	Separated/Divorced	4	1.2
Religion	Catholic	233	69.3
	Christian (non-Catholic)	72	21.4
	Other	23	6.8
	Jehovah’s Witness	7	2.1
	Mormon	1	0.3
Socioeconomic stratum*	0	7	2.1
	1	67	19.9
	2	172	51.2
	3	83	24.7
	4	6	1.8
	5	1	0.3
Education level	Incomplete primary	117	34.8
	Complete primary	73	21.7
	Incomplete secondary	67	19.9
	Complete secondary	53	15.8
	Technical	18	5.4
	University	8	2.4
Lives with family	Yes	288	85.7
	No	48	14.3
Household size	Lives alone	38	11.3
	1 person	77	22.9
	2 people	71	21.1
	3 people	53	15.8
	4 people	47	14.0
	5 people	26	7.7
	>5 people	24	7.1
Relationship with cohabitants	Poor	3	0.9
	Fair	24	7.1
	Good	270	80.4
	Not applicable	38	11.3
	No response	1	0.3
Member of a social group	No	293	87.2
	Yes	43	12.8
Receives support for treatment/illness	Yes	240	71.4
	No	96	28.6

### 3.2 Knowledge of T2DM self-care

**Figure 1:**
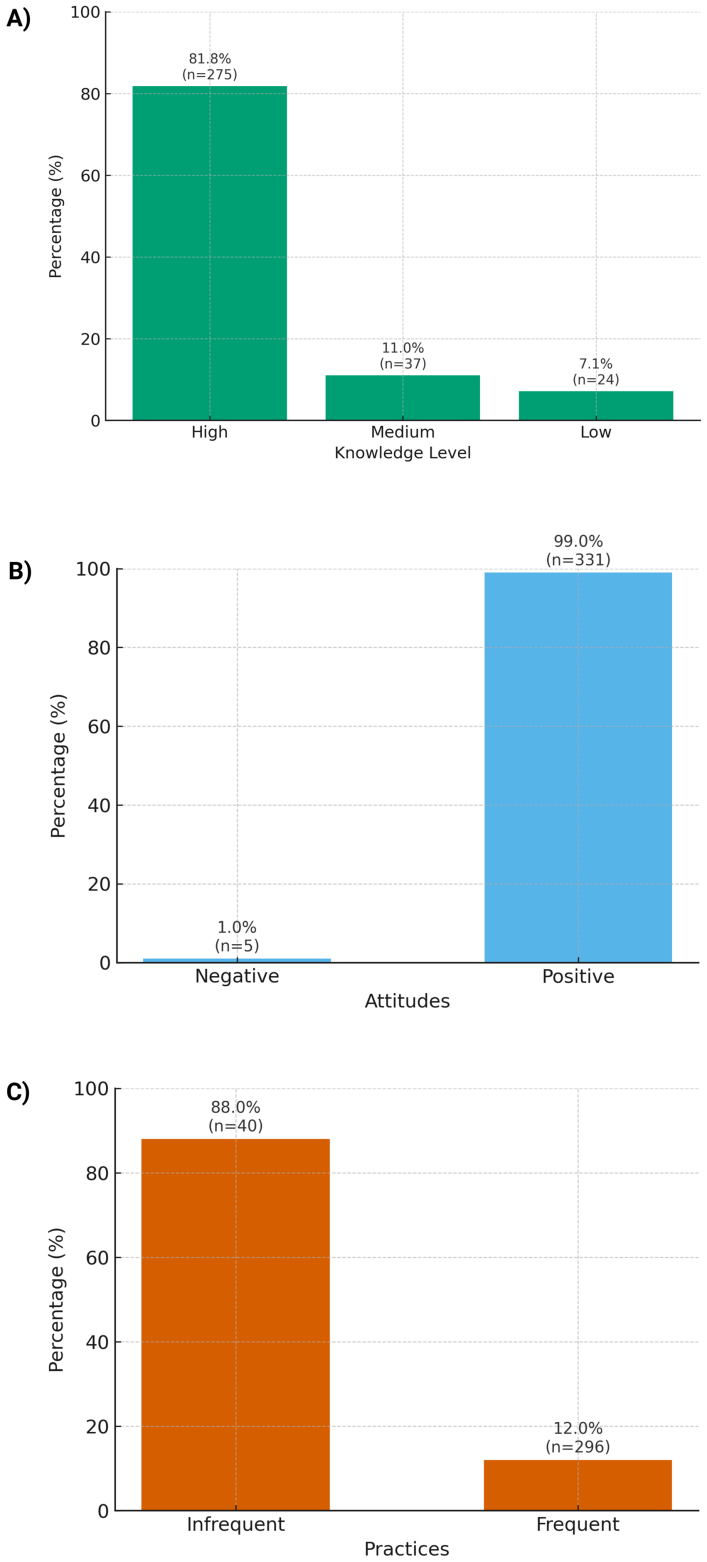
KAP profile of diabetes self-care among adults with type 2 diabetes in Cali, Colombia: **(A)** Knowledge level (high, medium, low), **(B)** attitudes (positive vs. negative), **(C)** practices (frequent vs. infrequent).

Overall, 81.8% of participants exhibited high knowledge (Figure 1A). Item-level gaps concentrated in daily meal planning (incorrect 73.5%; correct 26.5%) and understanding the value of self-monitoring of blood glucose (incorrect 68.2%; correct 31.8%). By contrast, recognition of clinical signs—such as linking polyuria and polydipsia to hypoglycemia—reached 57.1% correct (42.9% incorrect), and 67.0% correctly identified the appropriate frequency of medical follow-up (Supplementary Table 1).

#### 3.2.1 Variables associated with knowledge of T2DM self-care

Only socioeconomic stratum was significantly associated with knowledge level (*p* = 0.003). Strata 1–2 concentrated the highest proportions of high knowledge (85.1–85.5%), whereas strata 0 and 5 showed lower proportions; however, these extremes had very small cell sizes (*n* = 7 and *n* = 1), warranting cautious interpretation (Table 2). No significant associations were observed for the remaining variables (all *p* ≥ 0.179), with marginal trends for religion (*p* = 0.053) and household size (*p* = 0.085). No differences were found by sex, marital status, schooling, living with family, membership in a social group, or support for treatment (all *p* > 0.300) (Table 2).

**Table 2:** Sociodemographic and sociocultural factors associated with knowledge of self-care among patients with T2DM.

Variable		Total	Knowledge			*p*-value
		*n* (%)	Low *n* (%)	Medium *n* (%)	High *n* (%)	
Gender	Male	127 (37.8)	101 (79.5)	15 (11.8)	11 (8.7)	0.634
	Female	209 (62.2)	174 (83.3)	22 (10.5)	13 (6.2)	
Age (years)	30–59	115 (34.2)	91 (79.1)	13 (11.3)	11 (9.6)	0.179
	60–69	115 (34.2)	96 (83.5)	9 (7.8)	10 (8.7)	
	70–99	106 (31.5)	88 (83.0)	15 (14.2)	3 (2.8)	
Marital status	Single	143 (42.6)	11 (7.7)	14 (9.8)	118 (82.5)	0.526
	Married	90 (26.8)	10 (11.1)	9 (10.0)	71 (78.9)	
	Widowed	49 (14.6)	1 (2.0)	6 (12.2)	42 (85.7)	
	Cohabiting	50 (14.9)	2 (4.0)	8 (16.0)	40 (80.0)	
	Separated/Divorced	4 (1.2)	0 (0.0)	0 (0.0)	4 (100.0)	
Religion	Mormon	1 (0.3)	1 (100.0)	0 (0.0)	0 (0.0)	0.053
	Catholic	233 (69.3)	14 (6.0)	28 (12.0)	191 (82.0)	
	Christian (non-Catholic)	72 (21.4)	6 (8.3)	7 (9.7)	59 (81.9)	
	Other	23 (6.8)	2 (8.7)	2 (8.7)	19 (82.6)	
	Jehovah’s Witness	7 (2.1)	1 (14.3)	0 (0.0)	6 (85.7)	
Socioeconomic stratum†	0	7 (2.1)	2 (28.6)	1 (14.3)	4 (57.1)	**0.003**
	1	67 (19.9)	6 (9.0)	4 (6.0)	57 (85.1)	
	2	172 (51.2)	9 (5.2)	16 (9.3)	147 (85.5)	
	3	83 (24.7)	6 (7.2)	15 (18.1)	62 (74.7)	
	4	6 (1.8)	0 (0.0)	1 (16.7)	5 (83.3)	
	5	1 (0.3)	1 (100.0)	0 (0.0)	0 (0.0)	
Education level	Incomplete primary	117 (34.8)	6 (5.1)	9 (7.7)	102 (87.2)	0.418
	Complete primary	73 (21.7)	6 (8.2)	11 (15.1)	56 (76.7)	
	Incomplete secondary	67 (19.9)	3 (4.5)	8 (11.9)	56 (83.6)	
	Complete secondary	53 (15.8)	8 (15.1)	6 (11.3)	39 (73.6)	
	Technical	18 (5.4)	1 (5.6)	2 (11.1)	15 (83.3)	
	University	8 (2.4)	0 (0.0)	1 (12.5)	7 (87.5)	
Lives with family	No	48 (14.3)	4 (8.3)	8 (16.7)	36 (75.0)	0.357
	Yes	288 (85.7)	20 (6.9)	29 (10.1)	239 (83.0)	
Household size	Lives alone	38 (11.3)	4 (10.5)	6 (15.8)	28 (73.7)	0.085
	1 person	77 (22.9)	7 (9.1)	9 (11.7)	61 (79.2)	
	2 people	71 (21.1)	1 (1.4)	12 (16.9)	58 (81.7)	
	3 people	53 (15.8)	4 (7.5)	2 (3.8)	47 (88.7)	
	4 people	47 (14.0)	5 (10.6)	2 (4.3)	40 (85.1)	
	5 people	26 (7.7)	3 (11.5)	1 (3.8)	22 (84.6)	
	>5 people	24 (7.1)	0 (0.0)	5 (20.8)	19 (79.2)	
Relationship with cohabitants	Not applicable	38 (11.3)	4 (10.5)	6 (15.8)	28 (73.7)	0.938
	Good	270 (80.4)	18 (6.7)	28 (10.4)	224 (83.0)	
	Fair	24 (7.1)	2 (8.3)	3 (12.5)	19 (79.2)	
	Poor	3 (0.9)	0 (0.0)	0 (0.0)	3 (100.0)	
	No response	1 (0.3)	0 (0.0)	0 (0.0)	1 (100.0)	
Member of a social group	No	293 (87.2)	19 (6.5)	32 (10.9)	242 (82.6)	0.458
	Yes	43 (12.8)	5 (11.6)	5 (11.6)	33 (76.7)	
Receives support for treatment/illness	No	96 (28.6)	8 (8.3)	11 (11.5)	77 (80.2)	0.846
	Yes	240 (71.4)	16 (6.7)	26 (10.8)	198 (82.5)	

Bold values indicate statistically significant results (*p* < 0.01).

### 3.3 Attitudes on T2DM self-care

Global attitude was markedly favorable: 99.0% positive (Figure 1B). At the item level, responses were strongly positive for willingness to change lifestyle (92.0% positive) and interest in learning self-care activities to prevent complications (87.0% positive). The least favorable aspect was the difficulty implementing lifestyle changes (only 11.0% positive), suggesting practical barriers despite the declared willingness (Supplementary Table 2).

#### 3.3.1 Variables associated with attitudes to T2DM self-care

In bivariate analyses, significant differences emerged by marital status (*p* = 0.001): positivity was nearly universal among the married and cohabiting (100%), slightly lower among the single (99.3%), and substantially lower among the widowed (91.8%). By religion, positive attitudes were very high among Catholics (98.7%) and non-Catholic Christians (100%) (*p <* 0.001). Regarding socioeconomic stratum, positivity was elevated in 2–4 (≥99.4–100%), with lower proportions in stratum 1 (95.5%) and in the single case in stratum 5 (0% positive); extreme strata again involved small cells (*p <* 0.001). Household size showed a gradient: households of 1–5 persons had 99–100% positive attitudes, whereas households with >5 cohabitants decreased to 83.3% (*p <* 0.001) (Table 3).

**Table 3:** Sociodemographic and sociocultural factors associated with attitudes to self-care among patients with T2DM.

Variable		Total	Attitudes		*p*-value
		*n* (%)	Negative *n* (%)	Positive *n* (%)	
Gender	Male	127 (37.8)	4 (3.1)	123 (96.9)	0.134
	Female	209 (62.2)	1 (0.5)	208 (99.5)	
Age (years)	30–59	115 (34.2)	1 (0.9)	114 (99.1)	0.179
	60–69	115 (34.2)	1 (0.9)	114 (99.1)	
	70–99	106 (31.5)	3 (2.8)	103 (97.2)	
Marital status	Single	143 (42.6)	1 (0.7)	142 (99.3)	0.001
	Married	90 (26.8)	0 (0.0)	90 (100.0)	
	Widowed	49 (14.6)	4 (8.2)	45 (91.8)	
	Cohabiting	50 (14.9)	0 (0.0)	50 (100.0)	
	Separated/Divorced	4 (1.2)	0 (0.0)	4 (100.0)	
Religion	Mormon	1 (0.3)	1 (100.0)	0 (0.0)	<0.001
	Catholic	233 (69.3)	3 (1.3)	230 (98.7)	
	Christian (non-Catholic)	72 (21.4)	0 (0.0)	72 (100.0)	
	Other	23 (6.8)	0 (0.0)	23 (100.0)	
	Jehovah’s Witness	7 (2.1)	1 (14.3)	6 (85.7)	
Socioeconomic stratum†	0	7 (2.1)	0 (0.0)	7 (100.0)	<0.001
	1	67 (19.9)	3 (4.5)	64 (95.5)	
	2	172 (51.2)	1 (0.6)	171 (99.4)	
	3	83 (24.7)	0 (0.0)	83 (100.0)	
	4	6 (1.8)	0 (0.0)	6 (100.0)	
	5	1 (0.3)	1 (100.0)	0 (0.0)	
Education level	Incomplete primary	117 (34.8)	1 (0.9)	116 (99.1)	0.771
	Complete primary	73 (21.7)	1 (1.4)	72 (98.6)	
	Incomplete secondary	67 (19.9)	1 (1.5)	66 (98.5)	
	Complete secondary	53 (15.8)	1 (1.9)	52 (98.1)	
	Technical	18 (5.4)	1 (5.6)	17 (94.4)	
	University	8 (2.4)	0 (0.0)	8 (100.0)	
Lives with family	No	48 (14.3)	2 (4.2)	46 (95.8)	0.312
	Yes	288 (85.7)	3 (1.0)	285 (99.0)	
Household size	Lives alone	38 (11.3)	1 (2.6)	37 (97.4)	<0.001
	1 person	77 (22.9)	0 (0.0)	77 (100.0)	
	2 people	71 (21.1)	0 (0.0)	71 (100.0)	
	3 people	53 (15.8)	0 (0.0)	53 (100.0)	
	4 people	47 (14.0)	0 (0.0)	47 (100.0)	
	5 people	26 (7.7)	0 (0.0)	26 (100.0)	
	>5 people	24 (7.1)	4 (16.7)	20 (83.3)	
Relationship with cohabitants	Not applicable	38 (11.3)	1 (2.6)	37 (97.4)	0.944
	Good	270 (80.4)	4 (1.5)	266 (98.5)	
	Fair	24 (7.1)	0 (0.0)	24 (100.0)	
	Poor	3 (0.9)	0 (0.0)	3 (100.0)	
	No response	1 (0.3)	0 (0.0)	1 (100.0)	
Member of a social group	No	293 (87.2)	3 (1.0)	290 (99.0)	0.246
	Yes	43 (12.8)	2 (4.7)	41 (95.3)	
Receives support for treatment/illness	No	96 (28.6)	2 (2.1)	94 (97.9)	0.943
	Yes	240 (71.4)	3 (1.2)	237 (98.8)	

### 3.4 Practices on T2DM self-care

At the global level, frequent self-care practices predominated (88.1%) (Figure 1C). At the item level, we observed solid performance for medication adherence (taking prescribed diabetes medication in the last 7 days: 76.0% frequent) and foot care (drying between toes after washing: 76.0% frequent). Risk behaviors were uncommon: recent smoking 4.0% and dessert/sweets consumption 19.0% in the last 7 days (Supplementary Table 3).

#### 3.4.1 Variables associated with self-care practices in T2DM

In bivariate associations, no statistically significant differences were identified between practices (frequent vs. infrequent) and the sociodemographic or sociocultural variables evaluated (all *p* > 0.050). Non-significant tendencies warranting exploration in future analyses were noted—for example, a higher proportion of frequent practices in stratum 3 (92.8%) versus stratum 1 (82.1%), and a slight variation by marital status (better performance among those living with a partner; *p* = 0.089). Overall, the findings suggest a favorable self-care profile in key behaviors (adherence and foot care) and low prevalence of harmful habits, without clear gradients by socioeconomic or cultural characteristics (Table 4).

**Table 4:** Sociodemographic and sociocultural factors associated with self-care practices among patients with T2DM.

Variable		Total	Practices		*p*-value
		*n* (%)	Infrequent *n* (%)	Frequent *n* (%)	
Gender	Male	127 (37.8)	112 (88.2)	15 (11.8)	0.999
	Female	209 (62.2)	184 (88.0)	25 (12.0)	
Age (years)	30–59	115 (34.2)	102 (88.7)	13 (11.3)	0.457
	60–69	115 (34.2)	98 (85.2)	17 (14.8)	
	70–99	106 (31.5)	96 (90.6)	10 (9.4)	
Marital status	Single	143 (42.6)	21 (14.7)	122 (85.3)	0.089
	Married	90 (26.8)	14 (15.6)	76 (84.4)	
	Widowed	49 (14.6)	4 (8.2)	45 (91.8)	
	Cohabiting	50 (14.9)	1 (2.0)	49 (98.0)	
	Separated/Divorced	4 (1.2)	0 (0.0)	4 (100.0)	
Religion	Mormon	1 (0.3)	0 (0.0)	1 (100.0)	0.888
	Catholic	233 (69.3)	28 (12.0)	205 (88.0)	
	Christian (non-Catholic)	72 (21.4)	9 (12.5)	63 (87.5)	
	Other	23 (6.8)	3 (13.0)	20 (87.0)	
	Jehovah’s Witness	7 (2.1)	0 (0.0)	7 (100.0)	
Socioeconomic stratum†	0	7 (2.1)	2 (28.6)	5 (71.4)	0.232
	1	67 (19.9)	12 (17.9)	55 (82.1)	
	2	172 (51.2)	20 (11.6)	152 (88.4)	
	3	83 (24.7)	6 (7.2)	77 (92.8)	
	4	6 (1.8)	0 (0.0)	6 (100.0)	
	5	1 (0.3)	0 (0.0)	1 (100.0)	
Education level	Incomplete primary	117 (34.8)	17 (14.5)	100 (85.5)	0.489
	Complete primary	73 (21.7)	8 (11.0)	65 (89.0)	
	Incomplete secondary	67 (19.9)	8 (11.9)	59 (88.1)	
	Complete secondary	53 (15.8)	7 (13.2)	46 (86.8)	
	Technical	18 (5.4)	0 (0.0)	18 (100.0)	
	University	8 (2.4)	0 (0.0)	8 (100.0)	
Lives with family	No	48 (14.3)	8 (16.7)	40 (83.3)	0.390
	Yes	288 (85.7)	32 (11.1)	256 (88.9)	
Household size	Lives alone	38 (11.3)	4 (10.5)	34 (89.5)	0.679
	1 person	77 (22.9)	5 (6.5)	72 (93.5)	
	2 people	71 (21.1)	11		

#### 3.4.2 Evaluation of the study’s methodological rigor

The study’s rigor was ensured through a cross-sectional design with simple random sampling and a statistically calculated sample size to guarantee representativeness. Validated instruments (DKQ-24, Likert scale, and adapted SDSCA) were used, piloted, and demonstrated adequate internal consistency (*α* > 0.7). Data collection was conducted through standardized interviews and parallel verification with institutional clinical records. Statistical analysis employed procedures appropriate for KAP studies, under ethical supervision approved by institutional committees. This set of elements ensures methodological quality, internal consistency, and validity in the interpretation of the findings.

#### 3.4.3 Methodological limitations of the study

Among the main limitations are the cross-sectional design, which prevents the establishment of causal relationships, and the use of self-reporting, which is susceptible to social desirability bias in self-care practices. Multivariate analyses were not performed, limiting the control of confounders. The completion rate relative to the sampling frame was low (22.9%), with possible participation bias. Furthermore, certain sociodemographic categories had small sample sizes, affecting the stability of the associations. Finally, the research was conducted in a single urban public network, which limits the generalizability of the results to other population contexts.

## 4 Discussion

This study characterizes the KAP profile of individuals with T2DM receiving care in an urban, first-level public health service in Cali, Colombia, and reveals a mixed pattern: high levels of knowledge (81.8%) and overwhelmingly positive attitudes (99.0%) co-occur with predominantly frequent practices (88.1%). Medication adherence and foot care reached satisfactory proportions, whereas specific gaps persist—particularly in meal planning and in understanding the value of self-monitoring—alongside a non-trivial share of recent sweets consumption (30–32). This contrast aligns with the literature on the “KAP gap,” whereby knowing and being willing do not always translate into sustained behavioral change in the most demanding components of day-to-day routines (30, 31).

Regarding social determinants, socioeconomic stratum was significantly associated with knowledge level, with better results in intermediate strata and worse at the extremes; however, given the small size of some cells, these differences should be interpreted cautiously (33, 34). This pattern is consistent with evidence on social gradients in health literacy and diabetes self-care (35, 36). In the attitudinal domain, associations were observed with marital status, religious affiliation, socioeconomic stratum, and household size. Widowhood and living in households with more than five people were linked to lower proportions of positive attitudes, suggesting potential emotional, support, or caregiving-burden barriers (37, 38); by contrast, cohabiting couples and households of one to five members showed near-universal acceptance (39). Variation by religiosity—with very high positivity among Catholics and non-Catholic Christians and lower positivity in minority categories—opens a window to partner with faith leaders and networks as allies in promoting culturally acceptable self-care (40, 41). In contrast, the practices domain did not show statistically significant associations with the variables assessed, although non-conclusive trends—e.g., better performance in stratum 3 and among those living with a partner—warrant confirmation using multivariable models (34, 42, 43).

From a programmatic perspective, the findings suggest that in urban contexts with socioeconomic vulnerability it is possible to achieve favorable knowledge and attitudes through primary-care strategies, but closing the distance between “knowing/wanting” and “doing” requires more finely tuned interventions (44, 45). Priority areas include strengthening content and support for dietary planning and physical activity, while sustaining gains in medication adherence and foot care. Working with families and faith communities, together with culturally safe materials, may enhance the pertinence and sustainability of messages—especially for subgroups with higher attitudinal risk, such as widowed individuals or very large households (33, 44, 45).

This study has notable methodological strengths, including the use of a structured instrument with demonstrated internal consistency (Cronbach’s alpha > 0.7 in all three domains), a clear operationalization of KAP categories defined *a priori*, and the integration of primary survey data with secondary clinical records to verify diagnosis and program enrollment The focus on a specific socio-historical context—users of a public primary care network in an urban Colombian setting—adds depth to the interpretation of social and cultural determinants that are seldom examined in self-care assessments (46, 47). Nevertheless, several limitations must be acknowledged. First, the cross-sectional design precludes establishing causal relationships between sociodemographic/sociocultural factors and KAP outcomes. Second, reliance on self-reported information introduces potential recall and social desirability bias, particularly for diet and physical activity. Third, the absence of multivariable analyses limits the ability to control for residual confounding and to disentangle the independent effects of overlapping social determinants. Fourth, the study was conducted in a single public primary care network, which may constrain the generalizability of the findings to other regions, rural settings, or private healthcare systems. In addition, the completion proportion relative to the sampling frame was 22.9%, and the number of individuals contacted was not recorded separately, raising the possibility of participation bias if those who agreed to participate differ systematically from non-respondents (48, 49).

Beyond these methodological considerations, there are also important contextual and conceptual limitations. Some potentially relevant contextual factors—such as the intensity and quality of diabetes education provided within the program, neighborhood-level conditions (e.g., safety, walkability), the actual affordability and availability of healthy foods, and specific economic or caregiving burdens—were not directly measured. These unmeasured variables may influence both KAP domains and the observed associations with sociodemographic and sociocultural characteristics, and should be explicitly incorporated into future studies. Furthermore, the study was not explicitly grounded in a formal behavioral or socio-ecological theoretical framework (e.g., Health Belief Model, Socio-Ecological Model), which limits the conceptual structure for interpreting the determinants of self-care. Embedding future research and intervention design within such frameworks could provide a more systematic understanding of how individual, interpersonal, community, and structural factors interact to shape diabetes self-management.

Overall, these findings support the premise that strengthening the continuity and quality of primary care, combined with context-sensitive diabetes self-management education, can enhance the impact of health systems on T2DM outcomes in urban Latin American settings (50). Future research should employ longitudinal designs to track changes in KAP over time, integrate mixed methods to capture patients’ lived experiences and perceived barriers, evaluate the role of contextual factors and psychosocial constructs such as self-efficacy and perceived support, and test culturally adapted interventions that specifically target the most lagging behavioral components of self-care (51, 52).

## 5 Conclusion

In an urban, primary care setting in Cali, Colombia, adults with T2DM exhibited a favorable KAP profile: high knowledge, overwhelmingly positive attitudes, and predominantly frequent self-care practices. Nevertheless, specific gaps persist in dietary planning and blood-glucose self-monitoring. Knowledge displayed a socioeconomic gradient (better performance in intermediate strata), whereas attitudes were less favorable among widowed individuals and those living in households with >5 members; no significant differences were observed for practices across the variables examined.

These findings support prioritizing targeted interventions that strengthen diet and self-monitoring while sustaining gains in medication adherence and foot care. Implementation should be anchored in family and community networks (including faith-based actors) and be culturally appropriate, with differentiated follow-up for more vulnerable subgroups (widowhood, large households, and socioeconomic extremes). Multivariable and longitudinal evaluations are needed to confirm these patterns and to assess the impact of tailored programs on lagging behavioral components.

## Data Availability

The original contributions presented in the study are included in the article/Supplementary material, further inquiries can be directed to the corresponding author.
