# Supplementary material for: A cross-sectional primary care study of knowledge, attitudes, and practices of type 2 diabetes self-care and their association with sociodemographic and sociocultural factors in Cali, Colombia
Source: Front Public Health. 2026 Jan 12;13:1719863. doi: 10.3389/fpubh.2025.1719863 (PMC12833467; doi:10.3389/fpubh.2025.1719863)
Supplement: Supplementary file 1 [file Table_1.DOCX]

**Supplementary Table 1.** Diabetes self-care knowledge items and response distribution (Correct vs. Incorrect; n=336).

| **Knowledge question** | **Response** | **n** | **%** |
| --- | --- | --- | --- |
| 1. If you have diabetes, how often should you see your doctor? | Incorrect | 111 | 33.0 |
|  | Correct | 225 | 67.0 |
| 2. Measuring your blood sugar helps you to… | Incorrect | 229 | 68.2 |
|  | Correct | 107 | 31.8 |
| 3. How often should you check your blood sugar? | Incorrect | 156 | 46.4 |
|  | Correct | 180 | 53.6 |
| 4. Frequent urination and thirst are signs of low blood sugar. | Incorrect | 144 | 42.9 |
|  | Correct | 192 | 57.1 |
| 5. If you notice increased thirst, more frequent urination, weight loss, sugar in the urine, and loss of appetite, what do you think is happening? | Incorrect | 182 | 54.2 |
|  | Correct | 154 | 45.8 |
| 6. What do you know about alternative treatments to improve diabetes (herbs, acupuncture, homeopathy, supplements)? | Incorrect | 106 | 31.5 |
|  | Correct | 230 | 68.5 |
| 7. How many meals per day should a person with diabetes consume? | Incorrect | 247 | 73.5 |
|  | Correct | 89 | 26.5 |

**Supplementary Table 2.** Attitudes toward diabetes self-care and treatment: Positive vs. Negative responses (n=336).

| **Attitude question** | **Response** | **n** | **%** |
| --- | --- | --- | --- |
| 1. What was your attitude when you were diagnosed with diabetes? | Negative | 179 | 53.0 |
|  | Positive | 157 | 47.0 |
| 2. Are you interested in learning self-care activities to prevent complications? | Negative | 44 | 13.0 |
|  | Positive | 292 | 87.0 |
| 3. Are you willing to change your lifestyle to control diabetes? | Negative | 26 | 8.0 |
|  | Positive | 310 | 92.0 |
| 4. Which of the following lifestyle changes has posed the greatest difficulty for controlling your diabetes? | Negative | 300 | 89.0 |
|  | Positive | 36 | 11.0 |
| 5. What was your reaction when you were prescribed treatment (pills or insulin)? | Negative | 265 | 79.0 |
|  | Positive | 71 | 21.0 |

**Supplementary Table 3.** Self-care practices in the previous seven days: Item-level frequency (Frequent vs. Infrequent; n=336).

| **Practice question** | **Response** | **n** | **%** |
| --- | --- | --- | --- |
| 1. In how many of the last seven days did you eat five or more servings of fruits and vegetables? | Infrequent | 172 | 51.0 |
|  | Frequent | 164 | 49.0 |
| 2. In how many of the last seven days did you include sweets or desserts in your meals? | Infrequent | 271 | 81.0 |
|  | Frequent | 65 | 19.0 |
| 3. In how many of the last seven days did you perform at least 30 minutes of physical activity (continuous activities such as walking)? | Infrequent | 207 | 62.0 |
|  | Frequent | 129 | 38.0 |
| 4. In how many of the last seven days did you engage in a specific exercise session (e.g., swimming, walking, cycling) beyond routine activities? | Infrequent | 224 | 67.0 |
|  | Frequent | 112 | 33.0 |
| 5. In how many of the last seven days did you perform capillary glucose testing as often as recommended by your physician? | Infrequent | 217 | 65.0 |
|  | Frequent | 119 | 35.0 |
| 6. In how many of the last seven days did you check your feet? | Infrequent | 119 | 35.0 |
|  | Frequent | 217 | 65.0 |
| 7. In how many of the last seven days did you dry between your toes after washing your feet? | Infrequent | 82 | 24.0 |
|  | Frequent | 254 | 76.0 |
| 8. In how many of the last seven days did you take your prescribed diabetes medications? | Infrequent | 79 | 24.0 |
|  | Frequent | 257 | 76.0 |
| 9. In how many of the last seven days did you take the recommended number of pills or insulin for diabetes? | Infrequent | 87 | 26.0 |
|  | Frequent | 249 | 74.0 |
| 10. Have you smoked a cigarette—even a single puff—during the last seven days? | Infrequent | 321 | 96.0 |
|  | Frequent | 15 | 4.0 |
| 11. When was the last time you smoked a cigarette? | Infrequent | 256 | 76.0 |
|  | Frequent | 80 | 24.0 |
